# Impact of sarcopenia on the prognosis of patients with advanced non‐small cell lung cancer treated with antiangiogenic therapy: A propensity score matching analysis

**DOI:** 10.1111/1759-7714.15443

**Published:** 2024-09-22

**Authors:** Fuchun Huang, Mingxuan Ma, Liye Lang, Shuang Yang, Hui Zhao, Jialin Zhang, Hua Liu

**Affiliations:** ^1^ Department of Pulmonary and Critical Care Medicine Affiliated Hospital of Nantong University, Medical School of Nantong University Nantong China

**Keywords:** antiangiogenic therapy, non‐small cell lung cancer, prognosis, propensity score matching, sarcopenia

## Abstract

**Background:**

Limited information is available regarding the impact of sarcopenia on the prognosis of antiangiogenic therapy in individuals with advanced non‐small cell lung cancer (NSCLC). This study primarily sought to examine the prognostic significance of sarcopenia in individuals with advanced NSCLC undergoing antiangiogenic therapy.

**Methods:**

We retrospectively enrolled all patients who met the inclusion and exclusion criteria from 2019 to 2021 at Nantong University Hospital. Patients were grouped according to the presence or absence of sarcopenia. After propensity score matching (PSM), progression‐free survival (PFS), overall survival (OS), and adverse event rates were compared between the two groups. Factors associated with prognosis were screened using univariate and multivariate analyses.

**Results:**

A total of 267 patients were included, with a total of 201 matched at baseline after PSM (77 in the sarcopenia group and 124 in the non‐sarcopenia group). The sarcopenia group had lower PFS (*p* = 0.043) and OS (*p* = 0.011) than the non‐sarcopenia group and a higher incidence of adverse events (*p* = 0.044). Multivariate analysis suggested that sarcopenia is an independent prognostic risk factor for OS in advanced NSCLC patients receiving antiangiogenic therapies (*p* = 0.009). Results of subgroup analyses showed some differences in the impact of sarcopenia on survival prognosis in populations with different characteristics.

**Conclusion:**

Patients with advanced NSCLC with comorbid sarcopenia exhibit a worse prognosis when treated with antiangiogenic therapy, and preventing and ameliorating sarcopenia may lead to better survival outcomes in patients with advanced NSCLC.

## Graphical abstract

Patients with advanced non‐small cell lung cancer who underwent antiangiogenic therapy were collected according to the inclusion and exclusion criteria. Image analysis software was used to diagnose whether patients had combined sarcopenia. Baseline parameters were adjusted for both groups of patients using propensity score matching. To compare the differences in survival time and incidence of adverse events between the two groups of patients before and after propensity score matching and to explore the risk factors affecting patient prognosis.

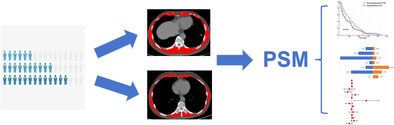

## INTRODUCTION

Lung cancer is a malignant tumor with high morbidity and mortality rates.^1^ In 2020, there were approximately 2.2 million new cases of lung cancer and 1.8 million lung cancer deaths. Of these, non‐small cell lung cancer (NSCLC) accounts for about 85% of all lung cancer patients.^2^ For recurrent or advanced NSCLC, in addition to chemotherapy, radiotherapy, molecularly targeted therapies, and immune checkpoint inhibitors, anti‐vascular therapy is also an important treatment modality. Combining with other treatments has a synergistic effect and significantly improves survival.^3^, ^4^, ^5^ However, it is not known whether all patients will have the same benefit.

Sarcopenia is a clinical syndrome characterized by decreased muscle size, decreased muscle strength, and/or reduced muscle function.^6^ Some scholars have found that sarcopenia is closely related to digestive tract tumors,^7^, ^8^ lung tumors,^9^, ^10^ breast cancer,^11^ lymphoma,^12^ and so on. Patients with tumors combined with sarcopenia tend to show poorer treatment tolerance and worse long‐term prognosis. Previous studies have shown that patients with tumors combined with sarcopenia exhibit a worse prognosis and more adverse events when treated with surgery,^13^ chemotherapy,^14^ targeted or immunotherapy.^15^ A study by Tsavachidou et al. found that among 37 patients with renal cell carcinoma treated with antiangiogenic therapy, the sarcopenia group demonstrated lower progression‐free survival (PFS) and overall survival (OS).^16^

However, it is unclear whether sarcopenia is an adverse risk factor for prognosis in patients with advanced lung cancer receiving antiangiogenic therapy. Therefore, the objective of this study was to explore the impact of skeletal sarcopenia on the prognosis of antiangiogenic therapy in advanced NSCLC.

## METHODS

### Study design and patients

The study was retrospective and reviewed by the Ethics Committee of Nantong University Hospital (No. 2024‐K007‐01). To secure an ample follow‐up duration, the present study incorporated individuals with advanced or recurrent NSCLC undergoing antiangiogenic therapy between January 2019 and December 2021 at the Affiliated Hospital of Nantong University.

Patients who met the following criteria were included: (1) patients with advanced or recurrent NSCLC clearly diagnosed as inoperable; (2) receiving antiangiogenic therapy for the first time and treated for at least two cycles; (3) received chest computed tomography (CT) within 4 weeks prior to antiangiogenic therapy; and (4) complete clinicopathological data, laboratory tests, and follow‐up data.

Patients meeting the following criteria were excluded: (1) advanced or recurrent NSCLC without definite pathological diagnosis; (2) combined with other malignant tumors or serious hematological or immune system diseases; (3) serious infections, rheumatic diseases, or other chronic diseases that seriously affect the results of laboratory tests within the last 1 month; and (4) those with missing clinical data or imaging data.

### Data collection and outcome indicators

We collected patients' basic information (gender, age, hypertension, diabetes, etc.), physical status (Karnofsky [KPS] score, Eastern Cooperative Oncology Group [ECOG] score, Nutritional Risk Screening [NRS‐2022] score), tumor‐related information (pathological type, stage, etc.), information on previous treatments, CT images, and other blood tests.

The study's main outcomes focused on PFS and OS, delineated as the duration from the commencement of antiangiogenic therapy to either disease progression or death, and the duration from the initiation of antiangiogenic therapy to eventual death, respectively. Secondary endpoints encompassed the overall response rate (ORR), disease control rate (DCR), and adverse events associated with antiangiogenic therapy. ORR was defined as the proportion of patients with a ≥30% reduction in tumor load from baseline that was maintained for more than 4 weeks after antiangiogenic therapy, which is the sum of the proportions of complete remission (CR) and partial remission (PR). DCR was defined as the proportion of patients who experienced tumor shrinkage or remained stable after antiangiogenic therapy, that is, the sum of the proportions of CR, PR, and stable disease (SD). Meanwhile, common adverse effects recorded from our collection of data include bleeding, hypertension, proteinuria, thromboembolism, skin toxicity, fatigue, elevated aminotransferases, abdominal pain, and generalized pain. Of these, bleeding, hypertension, proteinuria, and thromboembolism were grade 3 or higher adverse events.

### Diagnosis of sarcopenia

Typically, patients are determined to have sarcopenia by calculating the skeletal muscle index (SMI) at the third lumbar (L3) plane of the CT.^17^, ^18^ However, since patients with lung cancer usually undergo chest CT, L3 cannot be routinely visualized. According to previous studies, when L3 is not available, the tenth thoracic vertebrae level (T10) can be used instead of L3 to calculate SMI, and the diagnosis of sarcopenia can be made when the T10 SMI value is <28.8 cm^2^/m^2^ for men and <20.4 cm^2^/m^2^ for women.^19^

**FIGURE 1:**
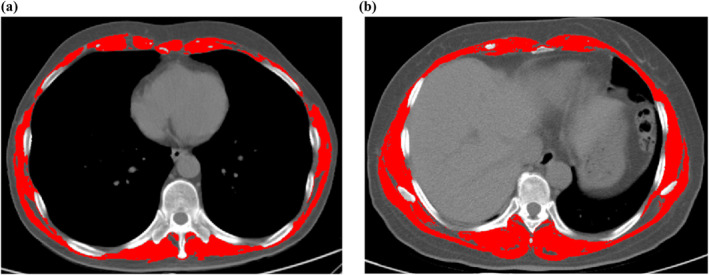
The red shaded part in the computed tomography image represents the area of skeletal muscle. (a) T10 skeletal muscle index (SMI) = 20.58 cm^2^/m^2^. (b) T10 SMI = 40.99 cm^2^/m^2^.

We utilized Hounsfield unit (HU) thresholding and employed SliceOma Version 5.0 (Tomovision, Montreal, QC, Canada) image analysis software for the quantification of the cross‐sectional area (cm^2^) of the T10 skeletal muscle.^20^ The HU range was set between −29 and 150 HU, and the muscles in the T10 plane were manually plotted and the skeletal muscle area (SMA) was calculated to derive the SMI using the height correction.^21^ A single example image representing these two different groups is that of a male with an SMI of 20.58 cm^2^/m^2^ classified as sarcopenia (Figure 1a), and a female with an SMI of 40.99 cm^2^/m^2^ classified as non‐sarcopenia (Figure 1b).

### Statistical analysis

Statistical analyses for this study were conducted utilizing IBM SPSS version 25.0 (SPSS Inc., Chicago, IL, USA). Normality tests were performed on the continuous variables (Table S1). For continuous variables adhering to a normal distribution, the *t*‐test was employed, presenting results as mean and standard deviation. Conversely, continuous variables exhibiting a skewed distribution underwent testing using the Mann–Whitney *U* test, with results expressed as median and range. For categorical variables, the *χ* ^2^ test or Fisher's exact test was used and the results were expressed as percentages and frequencies. To evaluate the prognosis of both groups, Kaplan–Meier survival curves were employed, with the log‐rank test utilized to assess distinctions between the two groups. Univariate and multivariate analyses were conducted using COX proportional hazard regression models to examine hazard ratios (HR) for both PFS and OS. A significance level of *p* < 0.05 was deemed statistically significant.

Due to the differences in baseline characteristics between the two groups of patients in the retrospective study, we performed PSM analyses applying a 1:2 nearest‐neighbor matching scheme with a caliper value of 0.2 in order to minimize the effects of bias in the subgroups.^22^

## RESULTS

### Baseline characteristics

**FIGURE 2:**
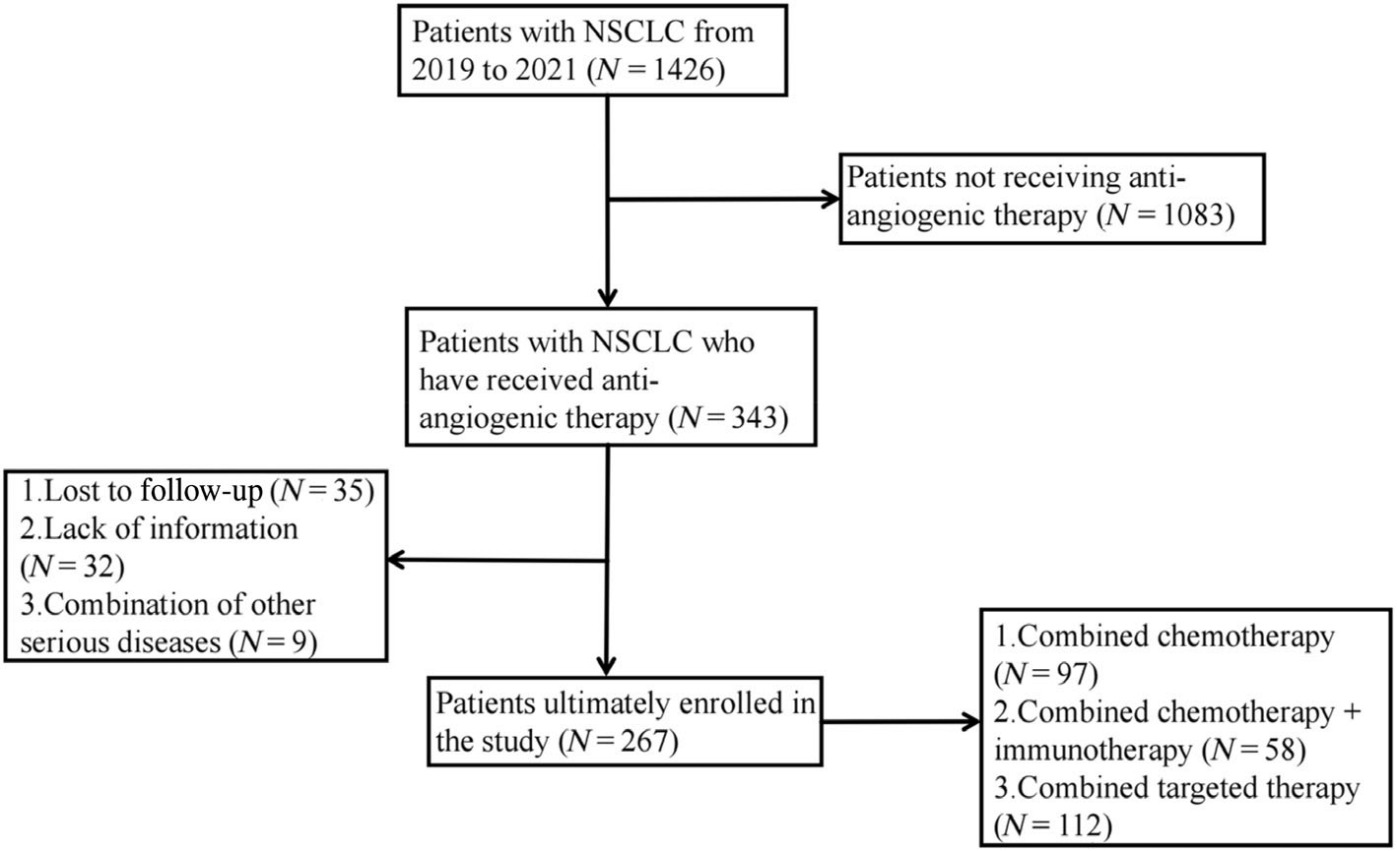
Flowchart of the screening process of patients finally included in this study. NSCLC, non‐small cell lung cancer.

The inclusion and exclusion criteria were strictly followed and finally a total of 267 patients were included in this study. Of note, all patients were receiving antiangiogenic therapy in combination with other treatment modalities. Of these, 97 patients were treated with combination chemotherapy, 58 patients were treated with combination chemotherapy and immunotherapy, and 112 patients were treated with combination targeted therapy (Figure 2). Two groups were divided according to the presence or absence of sarcopenia (sarcopenia group, *n* = 88; non‐sarcopenia group, *n* = 179). Significant differences were found in baseline characteristics of alkaline phosphatase, direct bilirubin, and lymphocyte count prior to pairing. After matching, 77 patients in the sarcopenia group and 124 patients in the non‐sarcopenia group (caliper = 0.2) were matched and all were balanced at baseline. Table 1 shows the baseline characteristics of the two groups of patients before and after PSM.

**TABLE 1:** Baseline characteristics of patients in the sarcopenia and non‐sarcopenia groups before and after propensity score matching.

Characteristics	Before matching (*N* = 267)		*p* Value	After 1:2 matching (*N* = 201)		*p* Value
	Sarcopenia group (*N* = 88)	Non‐sarcopenia group (*N* = 179)		Sarcopenia group (*N* = 77)	Non‐sarcopenia group (*N* = 124)	
Age (years)^a^	67.20 ± 8.77	61.98 ± 9.39	0.540	66. 18 ± 8.67	64.49 ± 8.74	0.181
ALT (IU/L)^b^	20.5 (14, 30)	19 (14, 28)	0.408	19 (13, 27.5)	21 (14, 30)	0.576
AST (IU/L)^b^	24.5 (20, 36)	24 (20, 31)	0.343	24 (19, 32)	26 (21, 32)	0.302
ALP (IU/L)^b^	91 (74, 116.25)	82 (65, 111)	0.046*	88 (70.5, 110.5)	84 (66, 111)	0.464
LDH (IU/L)^b^	225 (188.25, 306)	242 (193, 314)	0.451	216 (183.5, 284)	241 (191, 309)	0.182
Total bilirubin (μmol/L)^b^	9.45 (7.63, 13.35)	9.5 (7. 1, 12.3)	0.383	9.5 (7.85, 3. 15)	9.9 (7.2, 13. 1)	0.614
Direct bilirubin (μmol/L)^b^	2.95 (1.73, 4.30)	2.3 (1.4, 3.2)	0.008*	2.6 (1.7, 4. 1)	2.4 (1.7, 3.7)	0.299
D‐dimer (mg/L)^b^	0.76 (0.45, 1.36)	0.76 (0.43, 1.65)	0.937	0.75 (0.45, 1.33)	0.76 (0.44, 1.65)	0.896
PT (s)^b^	11.5 (11, 12.4)	11.4 (10.8, 11.9)	0.054	11.5 (11, 12.5)	11.4 (10.7, 12. 1)	0.072
APTT (s)^b^	28.25 (27.03, 29.35)	28. 1 (26.5, 29.6)	0.260	28.3 (26.9, 29.55)	28 (26.5, 29.3)	0.199
BUN (mmol/L)^b^	6. 1 (4.9, 7.2)	5.6 (4.6, 6.9)	0.176	6. 1 (4.9, 7.05)	5.8 (4.7, 7.2)	0.935
Cr (μmol/L)^b^	68 (60.5, 81.25)	67 (56, 75)	0.164	69 (61, 78.5)	69 (59, 78)	0.521
WBC (10^9^/L)^b^	5.9 (4.5, 7.4)	5.8 (4.8, 7.6)	0.751	5.6 (4.35, 7.3)	5.9 (4.8, 7.7)	0.168
Hemoglobin (g/L)^a^	118.0 ± 19.4	120.8 ± 18.6	0.735	119.03 ± 19.59	119.98 ± 18.47	0.742
PLT (10^9^/L)^b^	195.5 (148.5, 247.8)	210 (168, 262)	0.068	196 (147.5, 240)	200 (160, 263)	0.107
Neutrophils (10^9^/L)^b^	3.97 (2.83, 5. 15)	3.66 (2.76, 5. 13)	0.609	3.54 (2.64, 4.92)	3.69 (2.76, 5. 17)	0.534
Lymphocyte (10^9^/L)^b^	1. 18 (0.86, 1.51)	1.35 (1.02, 1.57)	0.023*	1. 18 (0.87, 1.54)	1.33 (0.93, 1.56)	0.170
NLR^b^	3.33 (2. 12, 4.92)	2.81 (2.02, 4.42)	0.053	3. 12 (2.07, 4.35)	2.94 (2.05, 5.06)	0.644
PLR^b^	164.44 (116.71, 224.67)	164. 10 (114.76, 227.43)	0.880	160.63 (114.68, 210. 14)	165.97 (111.68, 241.01)	0.675
Total cholesterol (mmol/L)^b^	4.2 (3.6, 4.9)	4.3 (3.7, 4.8)	0.765	4.2 (3.5, 4.8)	4.3 (3.8, 4.8)	0.378
Triglycerides (mmol/L)^b^	1.21 (0.92, 1.70)	1.37 (0.96, 1.71)	0.374	1.2 (0.92, 1.62)	1.37 (0.94, 1.74)	0.367
HDL (mmol/L)^b^	1.08 (0.85, 1.27)	1.06 (0.91, 1.26)	0.789	1.09 (0.86, 1.26)	1.06 (0.92, 1.25)	0.951
LDL (mmol/L)^a^	2.77 ± 0.77	2.74 ± 0.71	0.160	2.74 ± 0.79	2.79 ± 0.67	0.720
Albumin (g/L)^a^	38.50 ± 5.36	39.24 ± 5.06	0.632	38.92 ± 5.20	38.96 ± 5.13	0.975
Globulin (g/L)^b^	29.55 (27.00, 34.35)	29.3 (26.8, 32.6)	0.407	29.7 (27, 34.3)	29.5 (26.4, 32.2)	0.252
A/G^b^	1.25 (1.07, 1.48)	1.33 (1. 14, 1.47)	0.169	1.25 (1.08, 1.51)	1.33 (1.13, 1.46)	0.365
Serum CEA (ng/mL)^b^	9.50 (4.20, 45.53)	8.75 (3.18, 39.08)	0.532	7.9 (4.05, 43.9)	8.3 (3. 1, 35.2)	0.611
Gender (male), *n* (%)			0.178			0.692
Male	61 (69.3%)	109 (60.9%)		53 (68.8%)	82 (66. 1%)	
Female	27 (30.7%)	70 (39. 1%)		24 (31.2%)	42 (33.9%)	
Smoking history, *n* (%)			0.602			0.649
Yes	8 (9. 1%)	20 (11.2%)		6 (7.8%)	12 (9.7%)	
No	80 (90.9%)	159 (88.8%)		71 (92.2%)	112 (90.3%)	
Hypertension, *n* (%)			0.272			0.667
Yes	24 (27.3%)	38 (21.2%)		22 (28.6%)	32 (25.8%)	
No	64 (72.7)	141 (78.8)		55 (71.4)	92 (74.2%)	
Diabetes, *n* (%)			0.709			0.489
Yes	7 (8.0%)	12 (6.7%)		7 (9. 1%)	8 (6.5%)	
No	81 (92.0%)	167 (93.3%)		70 (90.9)	116 (93.5%)	
Pleural effusion, *n* (%)			0.904			0.968
Yes	41 (46.6%)	82 (45.8%)		35 (45.5%)	55 (45.2%)	
No	47 (53.4%)	97 (54.2%)		42 (54.5%)	69 (54.8%)	
ECOG score, *n* (%)			0.510			0.715
≥2	9 (10.2%)	14 (7.8%)		4 (5.2%)	8 (6.5%)	
0 ~ 1	79 (89.8%)	165 (92.2%)		73 (94.8%)	116 (93.5%)	
KPS score, *n* (%)			0.205			0.071
<60	3 (3.4%)	1 (0.6%)		2 (2.6%)	0 (0%)	
≥60	85 (96.6%)	178 (99.4%)		75 (97.4)	124 (100%)	
Urine protein, *n* (%)			0.887			0.580
Yes	1 (1. 1%)	4 (2.2%)		1 (1.3%)	3 (2.4%)	
No	87 (98.9%)	175 (97.8%)		76 (98.7%)	121 (97.6%)	
BMI (kg/m^2^), *n* (%)			0.458			0.556
≥24	26 (29.5%)	61 (34. 1%)		23 (29.9%)	42 (33.9%)	
<24	62 (70.5%)	118 (65.9%)		54 (70. 1%)	82 (66. 1%)	
NRS‐2002 score, *n* (%)			0.137			0.539
≥3	18 (20.5%)	24 (13.4%)		13 (16.9%)	17 (13.7%)	
<3	70 (79.5%)	155 (86.6%)		64 (83. 1%)	107 (86.3%)	
Pathological type			0.086			0.299
Adenocarcinoma, *n* (%)	66 (75.0%)	150 (83.8%)		58 (75.3%)	101 (81.5%)	
Non‐adenocarcinoma, *n* (%)	22 (25.0%)	29 (16.2%)		19 (24.7%)	23 (18.5%)	
TNM stage			0.468			0.661
IIIa, *n* (%)	3 (3.4%)	6 (3.4%)		3 (3.9%)	5 (4.0%)	
IIIb, *n* (%)	4 (4.5%)	3 (1.7%)		3 (3.9%)	3 (2.4%)	
IIIc, *n* (%)	0 (0.0%)	2 (1. 1%)		0 (0.0%)	2 (1.6%)	
IV, *n* (%)	81 (92.0%)	168 (93.9%)		71 (92.2%)	114 (91.9%)	
Previous treatment						
Chemotherapy, *n* (%)	78 (88.6%)	157 (87.7%)	0.826	70 (90.9%)	107 (86.3%)	0.326
Targeted therapy, *n* (%)	24 (27.3%)	69 (38.5%)	0.069	23 (29.9%)	47 (37.9%)	0.245
Immunotherapy, *n* (%)	29 (33.0%)	47 (26.3%)	0.254	27 (35. 1%)	38 (30.6%)	0.515
Radiotherapy, *n* (%)	3 (3.4%)	11 (6. 1%)	0.346	3 (3.9%)	9 (7.3%)	0.328
Surgical treatment, *n* (%)	11 (12.5%)	25 (14.0%)	0.742	10 (13%)	19 (15.3%)	0.647

*Note*: Categorical variables are presented as frequency (percentage) unless otherwise stated.

Abbreviations: ALP, alkaline phosphatase; ALT, alanine aminotransferase; APTT, activated partial thromboplastin time; AST, aspartate aminotransferase; BMI, body mass index; BUN, blood urea nitrogen; CEA, carcinoembryonic antigen; ECOG, Eastern Cooperative Oncology Group; HDL, high density lipoprotein; KPS, Karnofsky score; LDH, lactate dehydrogenase; LDL, low density lipoprotein; NLR, neutrophil lymphocyte ratio; NRS, Nutritional Risk Screening score; PLR, platelet‐lymphocyte ratio; PLT, blood platelet; PT, prothrombin time; TNM, Tumor Node Metastasis.

^a^ Continuous variables with normal distribution are presented as mean value ± standard deviation.

^b^ Others are presented as median (interquartile range).

* *p* < 0.05.

### Clinial outcomes

**FIGURE 3:**
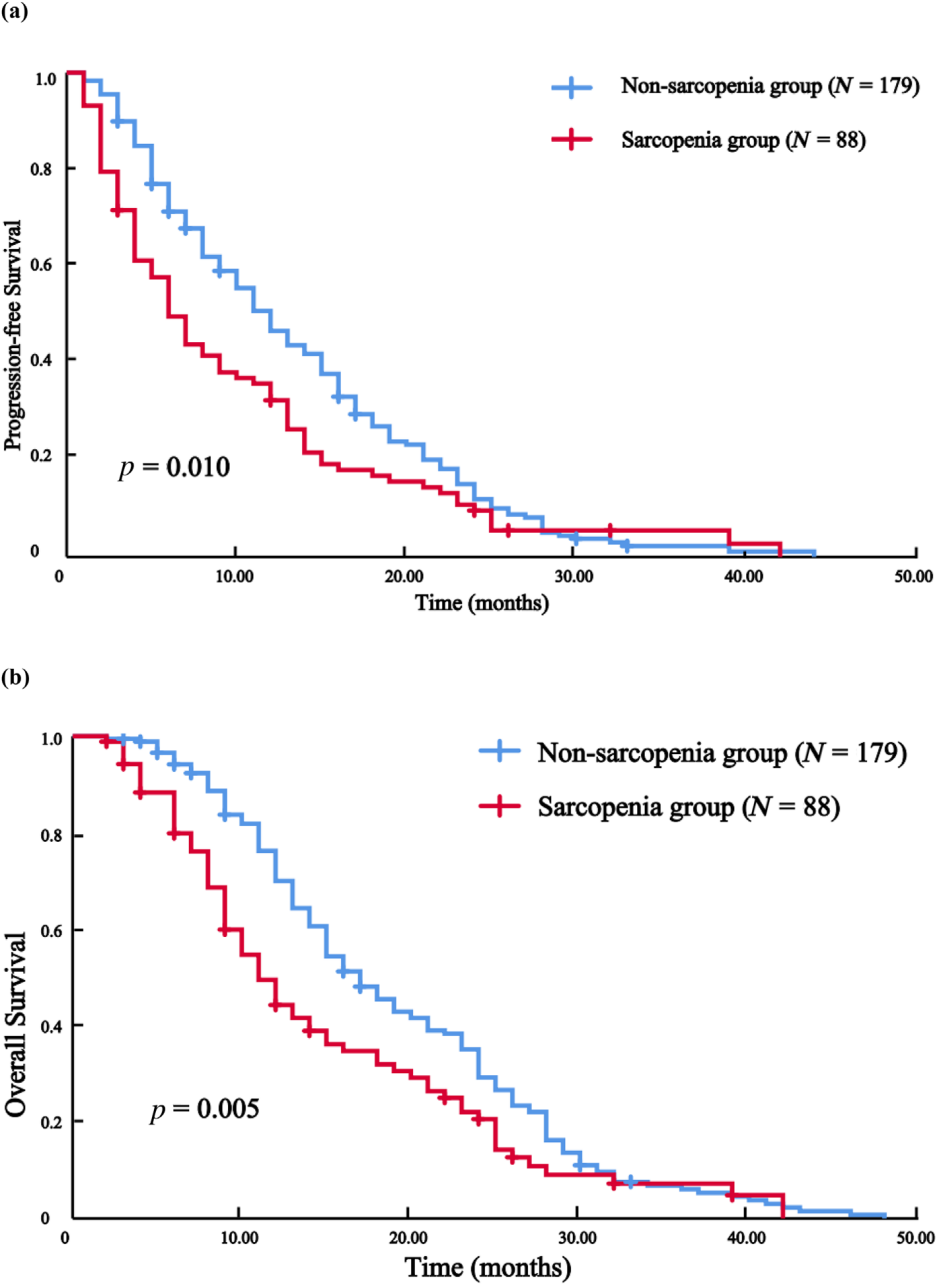
Before propensity score matching: Progression‐free survival (PFS) and overall survival (OS) of patients in the sarcopenia and non‐sarcopenia groups (*N* = 267). (a) PFS. (b) OS.

**FIGURE 4:**
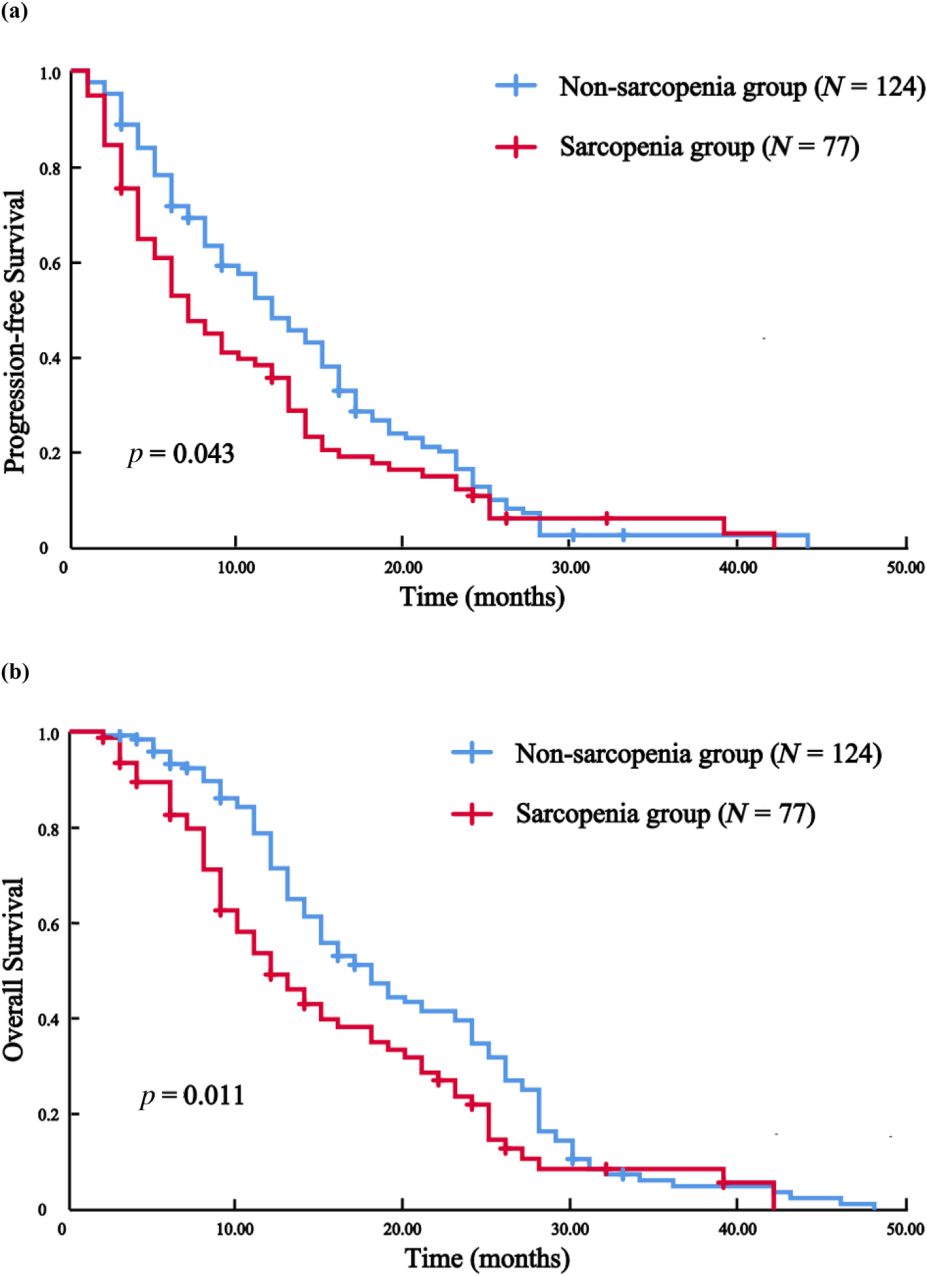
After propensity score matching: Progression‐free survival (PFS) and overall survival (OS) of patients in the skeletal sarcopenia and non‐skeletal sarcopenia groups (*N* = 201). (a) PFS. (b) OS.

The median duration of follow‐up was 15 months. Prior to PSM, the sarcopenia group exhibited median PFS and OS of 6 and 11 months, respectively. These values were significantly shorter than those observed in the normal group, which had median PFS and OS of 12 and 17 months, respectively (Figure 3a, *p* = 0.010; Figure 3b, *p* = 0.005). After PSM, median PFS and OS were 7 and 12 months in the sarcopenia group, compared with 12 and 18 months in the normal group, with significant differences between the two groups (Figure 4a, *p* = 0.043; Figure 4b, *p* = 0.011). As shown in Table 2, the incidence of total adverse events in patients in the sarcopenia group was 27.3% before PSM and 26.0% after PSM, which was much higher than that in the normal group, which was 12.3% (*p* = 0.002) and 14.5% (*p* = 0.044), respectively. In contrast, there was no significant difference between the two groups of patients before and after PSM in terms of objective remission rate, disease control rate, and incidence of adverse events of grade 3 or higher.

**TABLE 2:** Clinial outcome of patients in the sarcopenia and non‐sarcopenia groups before and after propensity score matching.

Characteristics	Before matching (*N* = 267)		*p* Value	After 1:2 matching (*N* = 201)		*p* Value
	Sarcopenia group (*N* = 88)	Non‐sarcopenia group (*N* = 179)		Sarcopenia group (*N* = 77)	Non‐sarcopenia group (*N* = 124)	
CR	0 (0%)	0 (0%)		0 (%)	0 (0%)	
PR	6 (6.8%)	12 (6.7%)	0.972	6 (7.8%)	10 (8. 1%)	0.945
SD	17 (19.3%)	52 (29. 1%)	0.088	17 (22. 1%)	39 (31.5%)	0.150
PD	65 (73.9%)	115 (64.2%)	0.115	54 (70. 1%)	75 (60.5%)	0.166
ORR (CR + PR)	6 (6.8%)	12 (6.7%)	0.972	6 (7.8%)	10 (8. 1%)	0.945
DCR (CR + PR + SD)	23 (26. 1%)	64 (35.8%)	0.115	23 (29.9%)	49 (39.5%)	0.166
Total adverse events	24 (27.3%)	22 (12.3%)	0.002*	20 (26.0%)	18 (14.5%)	0.044*
Adverse events ≥level 3	9 (10.2%)	17 (9.5%)	0.850	8 (10.4%)	14 (11.3%)	0.842
Hypertension	0 (0%)	3 (1.7%)	0.222	0 (%)	2 (1.6%)	0.263
Proteinuria	6 (6.8%)	5 (2.8%)	0.120	6 (7.8%)	4 (3.2%)	0.148
Bleeding	2 (2.3%)	9 (5%)	0.287	1 (1.3%)	7 (5.6%)	0.125
Thromboembolism	1 (1. 1%)	1 (0.6%)	0.607	1 (1.3%)	1 (0.8%)	0.732

*Note*: Categorical variables are presented as frequency (percentage) unless otherwise stated.

Abbreviations: CR, complete remission; DCR, disease control rate; ORR, overall response rate; PD, progressive disease; PR, partial remission; SD, stable disease.

* *p* < 0.05.

### Univariate and multivariate analyses

Univariate analyses showed that 17 and 14 variables exhibited differences in PFS and OS for patients in the sarcopenia and normal groups, respectively. Inclusion of these variables in a multivariate COX regression analysis showed that hemoglobin (HB) and KPS score were risk factors for PFS and OS. Skeletal sarcopenia was an independent risk factor for OS but not for PFS (*p* = 0.009 and 0.119) (Table 3).

**TABLE 3:** Univariate and multivariate analyses of each factor's value in predicting progression‐free survival (PFS) and overall survival (OS) of the whole study population.

	PFS				OS			
	Univariate analyses		Multivariate analyses		Univariate analyses		Multivariate analyses	
	HR (95% CI)	*p* Value	HR (95% CI)	*p* Value	HR (95% CI)	*p* Value	HR (95% CI)	*p* Value
Age (years)	1.72 (0.93–3. 17)	0.083			1.02 (0.54–1.91)	0.951		
ALT (IU/L)	0.86 (0.73–1.02)	0.075			0.81 (0.68–0.97)	0.020*	0.99 (0.98–1.00)	0.172
AST (IU/L)	0.86 (0.65–1. 14)	0.293			0.76 (0.56–1.04)	0.082		
ALP (IU/L)	1.28 (1.04–1.58)	0.022*	1.00 (1.00–1.00)	0.303	1.27 (1.01–1.60)	0.040*	1.00 (1.00–1.01)	0.239
LDH (IU/L)	0.90 (0.75–1.09)	0.298			0.82 (0.66–1.01)	0.062		
Total bilirubin (μmol/L)	0.9 (0.70–1. 16)	0.425			0.90 (0.69–1. 18)	0.462		
Direct bilirubin (μmol/L)	1.02 (0.86–1.21)	0.850			1.05 (0.87–1.25)	0.620		
D‐dimer (mg/L)	1.14 (0.98–1.33)	0.097			1.22 (1.04–1.45)	0.017*	1.01 (0.96–1.07)	0.741
PT (s)	4.77 (1.81–12.59)	0.002*	1.11 (0.95–1.29)	0.193	7.72 (2.65–22.47)	<0.001*	1.16 (0.99–1.36)	0.062
APTT (s)	0.75 (0.24–2.36)	0.626			0.54 (0.16–1.90)	0.339		
BUN (mmol/L)	0.93 (0.72–1.21)	0.607			0.83 (0.61–1.13)	0.236		
Cr (μmol/L)	1.16 (0.82–1.64)	0.414			0.95 (0.65–1.39)	0.801		
WBC (10^9^/L)	1.36 (0.98–1.89)	0.070			1.36 (0.96–1.92)	0.084		
Hemoglobin (g/L)	0.32 (0.18–0.57)	<0.001*	0.99 (0.98–1.00)	0.018*	0.33 (0.18–0.62)	<0.001*	0.99 (0.98–1.00)	0.047*
PLT (10^9^/L)	0.96 (0.78–1.18)	0.703			0.92 (0.74–1.16)	0.492		
Neutrophils (10^9^/L)	1.37 (1.06–1.77)	0.015*	1.08 (1.00–1.17)	0.061	1.42 (1.07–1.87)	0.014*	1.07 (0.98–1.16)	0.113
Lymphocyte (10^9^/L)	0.52 (0.35–0.77)	0.001*	0.80 (0.58–1.11)	0.178	0.51 (0.33–0.78)	0.002*	0.80 (0.57–1.11)	0.184
NLR	1.38 (1.18–1.61)	<0.001*	0.98 (0.93–1.02)	0.326	1.49 (1.25–1.77)	<0.001*	0.99 (0.94–1.04)	0.629
PLR	1.21 (1.03–1.44)	0.024*	1.00 (1.00–1.00)	0.915	1.22 (1.01–1.46)	0.035*	1.00 (1.00–1.00)	0.815
Total cholesterol (mmol/L)	1.31 (0.82–2.10)	0.260			1.15 (0.70–1.87)	0.584		
Triglycerides (mmol/L)	0.9 (0.67–1.20)	0.463			0.93 (0.69–1.27)	0.663		
HDL (mmol/L)	0.97 (0.54–1.76)	0.933			0.94 (0.53–1.67)	0.830		
LDL (mmol/L)	1.13 (0.76–1.69)	0.546			1.11 (0.73–1.69)	0.631		
Albumin (g/L)	0.39 (0.20–0.76)	0.006*	1.00 (0.97–1.03)	0.915	0.60 (0.28–1.27)	0.184		
Globulin (g/L)	0.82 (0.49–1.39)	0.463			0.72 (0.41–1.25)	0.241		
A/G	0.68 (0.33–1.41)	0.304			1.10 (0.51–2.37)	0.810		
Serum CEA (ng/mL)	1.06 (1.00–1.12)	0.045*	1.00 (1.00–1.00)	0.077	1.06 (1.00–1.12)	0.062		
TNM stage	0.54 (0.33–0.91)	0.019*	0.81 (0.64–1.02)	0.070	0.82 (0.45–1.48)	0.508		
Gender (male), *n* (%)								
Male versus female	1.44 (1.11–1.87)	0.006*	1.28 (0.96–1.73)	0.096	1.36 (1.04–1.79)	0.026*	1.25 (0.94–1.68)	0.131
Smoking history, *n* (%)								
With versus without	1.22 (0.81–1.82)	0.342			1.12 (0.74–1.69)	0.589		
Hypertension, *n* (%)								
With versus without	1.01 (0.75–1.36)	0.927			0.97 (0.71–1.32)	0.827		
Diabetes, *n* (%)								
With versus without	0.91 (0.54–1.53)	0.712			0.75 (0.41–1.37)	0.351		
Sarcopenia, *n* (%)								
With versus without	1.41 (1.08–1.83)	0.011*	1.25 (0.94–1.66)	0.119	1.48 (1.12–1.96)	0.006*	1.49 (1.10–2.00)	0.009*
Pleural effusion, *n* (%)								
With versus without	1.25 (0.97–1.61)	0.084			1.27 (0.97–1.65)	0.078		
ECOG score, *n* (%)								
0 ~ 1 versus ≥2	0.58 (0.37–0.90)	0.016*	0.78 (0.47–1.30)	0.343	0.78 (0.50–1.23)	0.290		
KPS score, *n* (%)								
<60 versus ≥60	3.70 (1.36–10.04)	0.010*	4.52 (1.39–14.71)	0.012*	4.75 (1.75–12.88)	0.002*	6.55 (2.26–18.93)	0.001*
Urine protein, *n* (%)								
With versus without	0.53 (0.20–1.42)	0.207			0.69 (0.26–1.87)	0.469		
BMI (kg/m^2^), *n* (%)								
≥24 versus <24	0.77 (0.59–1.01)	0.062			0.85 (0.65–1.13)	0.268		
NRS‐2002 score, *n* (%)								
≥3 versus <3	1.48 (1.06–2.07)	0.022*	1.30 (0.88–1.93)	0.182	1.45 (1.00–2.05)	0.049*	1.36 (0.91–2.05)	0.136
Adenocarcinoma, *n* (%)								
Yes versus no	0.84 (0.60–1.17)	0.295			1.10 (0.78–1.55)	0.598		
Chemotherapy, *n* (%)								
With versus without	1.44 (0.98–2.12)	0.061			1.49 (1.00–2.21)	0.051		
Targeted therapy, *n* (%)								
With versus without	0.76 (0.58–0.99)	0.039*	0.84 (0.62–1.13)	0.250	0.80 (0.61–1.05)	0.108		
Immunotherapy, *n* (%)								
With versus without	1.50 (1.14–1.99)	0.004*	1.11 (0.81–1.51)	0.519	1.52 (1.12–2.05)	0.007*	1.22 (0.88–1.70)	0.229
Radiotherapy, *n* (%)								
With versus without	0.53 (0.27–1.03)	0.062			0.70 (0.34–1.41)	0.313		
Surgical treatment, *n* (%)								
With versus without	0.94 (0.64–1.37)	0.733			1.01 (0.68–1.50)	0.956		
Total adverse events, *n* (%)								
With versus without	1.34 (0.97–1.61)	0.084			1.25 (0.89–1.76)	0.202		
Adverse events ≥level 3, *n* (%)								
With versus without	1.30 (0.86–1.97)	0.218			0.99 (0.64–1.52)	0.948		

Abbreviations: CI, confidence interval; ECOG, Eastern Cooperative Oncology Group; HR, hazard ratio; KPS, Karnofsky score; NRS, Nutritional Risk Screening score.

* *p* < 0.05.

### Subgroup analysis

Subgroups of patients with BMI ≥24 and BMI <24, ≥60 and <60 years of age, and with different combinations of treatment modalities were analyzed (Table 4). The results showed that in patients ≥60 years old, median PFS and median OS were 6 and 11 months in the sarcopenia group, which were significantly lower than those of 12 and 18 months in the normal group (*p* = 0.026 and 0.004). But the conclusion does not apply when the age is <60 years old. In patients with a BMI of <24 kg/m^2^, the median OS was significantly lower in the skeletal sarcopenia group (11 months) than in the normal group (17 months) (*p* = 0.011), whereas there was no significant difference in PFS. In addition, in overweight (BMI ≥ 24) patients, there was no significant difference in PFS and OS between the two groups. In patients treated with combination chemotherapy, the median PFS and OS in the sarcopenia group were 6 and 11 months, respectively, significantly lower than those in the normal group at 12 and 15 months (*p* = 0.010 and 0.029). In patients treated with combination chemotherapy and immunotherapy, the median OS was significantly lower in the sarcopenia group than in the normal group (10 vs. 14 months, *p* = 0.018), but there was no significant difference in median PFS between the two groups. However, in patients treated with targeted therapy in combination with antiangiogenic therapy, there was no significant difference in median PFS and OS between the two groups (*p* = 0.509 and 0.222).

**TABLE 4:** Subgroup analysis of 267 patients with advanced non‐small cell lung cancer.

Subgroup	Comorbidity with sarcopenia (no. of patients)	PFS (median PFS)	*p* Value	OS (median OS)	*p* Value
Age					
Age < 60	Sarcopenia (*n* = 18)	6 months	0.214	15 months	0.256
	Non‐sarcopenia (*n* = 69)	10 months		16 months	
Age ≥ 60	Sarcopenia (*n* = 70)	6 months	0.026*	11 months	0.004*
	Non‐sarcopenia (*n* = 110)	12 months		18 months	
BMI					
BMI < 24	Sarcopenia (*n* = 62)	6 months	0.084	11 months	0.011*
	Non‐sarcopenia (*n* = 118)	11 months		17 months	
BMI ≥ 24	Sarcopenia (*n* = 26)	8 months	0.101	15 months	0.197
	Non‐sarcopenia (*n* = 61)	13 months		18 months	
Combined treatment modalities					
Combined chemotherapy	Sarcopenia (*n* = 32)	6 months	0.010*	11 months	0.029*
	Non‐sarcopenia (*n* = 65)	12 months		15 months	
Combined chemotherapy + immunotherapy	Sarcopenia (*n* = 25)	6 months	0.149	10 months	0.018*
	Non‐sarcopenia (*n* = 33)	8 months		14 months	
Combined targeted therapy	Sarcopenia (*n* = 31)	12 months	0.509	15 months	0.222
	Non‐sarcopenia (*n* = 81)	15 months		21 months	

Abbreviations: BMI, body mass index; OS, overall survival; PFS, progression‐free survival.

* *p* < 0.05.

## DISCUSSION

Sarcopenia is an indicator of malnutrition and metabolic disorders and is caused by a variety of factors such as advanced age, neoplasia, malnutrition, lack of exercise, inflammatory diseases, and endocrine disorders.^23^, ^24^ Its impact on the survival prognosis of tumor patients has been confirmed by several studies.^25^, ^26^ Nakamura et al.^27^ found sarcopenia to be an independent poor prognostic factor in NSCLC patients after surgery. Buentzel et al. systematically reviewed the prognosis of lung cancer patients under multiple treatment modalities (surgical treatment, chemotherapy, targeted therapy, radiotherapy) and found that sarcopenia was significantly associated with shorter OS. Nevertheless, the influence of sarcopenia on antiangiogenic therapy outcomes in individuals with advanced NSCLC remains uncertain. In our investigation, NSCLC patients undergoing antiangiogenic therapy were categorized into the sarcopenia and normal groups. The findings indicated a notable reduction in both PFS and OS within the sarcopenia group compared to the normal group. In order to make our results more plausible, we performed PSM to make the baseline matching with the two groups of patients, and the results were consistent with before matching, suggesting that the prognostic differences may be more related to sarcopenia. We also looked at the occurrence of adverse events and found that sarcopenia was associated with more adverse events. The results of the multifactor regression similarly prove the above point. However, despite this, the results of subgroup analyses suggest that there may be differences in the effect of sarcopenia on survival time in populations with different characteristics. Rossi's study^28^ found that sarcopenia was a poor prognostic indicator of overall survival in patients with NSCLC receiving targeted therapy. However, this study found that sarcopenia had no significant effect on PFS and OS when antiangiogenic agents were combined with targeted therapy.

Therefore, we believe that the assessment of skeletal muscle mass and the prevention and improvement of sarcopenia in oncological patients is of particular importance. In previous studies, it is generally accepted that exercise interventions have a positive ameliorative effect on sarcopenia.^29^, ^30^, ^31^ Nutritional support may also reduce the incidence and slow the progression of sarcopenia.^32^, ^33^ Currently, there is no evidence that pharmacological interventions are effective, except for vitamin D and testosterone, which improve muscle mass to some extent.^34^, ^35^ In addition, Sánchez‐Lara's study found that NSCLC treated in the omega‐3 fatty acid group showed significant improvements in the degree of muscle mass loss and resistance compared to the control group.^36^ To enhance the unfavorable prognosis associated with sarcopenia, it is crucial to investigate supplementary interventions.

The underlying mechanisms regarding the impact of sarcopenia on the prognosis of NSCLC patients on antiangiogenic therapy are not fully understood. It has been suggested that it may be related to chronic inflammation in the body.^37^ It has also been suggested that IL‐6 and IL‐15 expressed in skeletal muscle cells are involved in the regulation of the body's immune system, and that low skeletal muscle mass leads to IL‐6 and IL‐15 deficiency, which affects the prognosis of tumors.^38^ In addition, the greater sensitivity of patients with sarcopenia to the toxicity of therapeutic agents may also contribute to the poor prognosis.^39^ In the future, we will conduct further studies to investigate the mechanisms by which sarcopenia contributes to the poor prognosis of NSCLC.

This study has the following shortcomings. Firstly, this is a retrospective study and it is difficult to avoid some bias affecting the results. Secondly, constrained by resource limitations, we could solely diagnose sarcopenia by computing the SMI value from CT scans; however, measurements of grip strength and step speed were not feasible. In addition, in real‐world studies, antiangiogenic therapy is not usually applied as a single therapy in the treatment of patients with advanced lung cancer, so the patients included in this study all applied other therapies while receiving antiangiogenic therapy, and in the future, we will use animal models to further investigate the effect of a single antiangiogenic therapy on the prognosis of lung cancer and its related mechanisms. Ultimately, this study was conducted at a single center with a limited sample size, thereby restricting the generalizability of the conclusions. More randomized controlled studies and large, multicenter, prospective cohort studies are needed to validate it.

## CONCLUSION

Sarcopenia may affect the efficacy of antiangiogenic therapy in patients with advanced NSCLC, reducing patients' PFS and OS and contributing more to adverse therapeutic events. Prevention and treatment of sarcopenia may improve the prognosis of patients with advanced NSCLC.

## AUTHOR CONTRIBUTIONS

Fuchun Huang and Mingxuan Ma were involved in the study design, data collection and analysis, interpretation of results, and drafting and revising the article. Shuang Yang, Hui Zhao, Jialin Zhang, and Liye Lang were involved in the collection and analysis of data and interpretation of results. Hua Liu participated in the revision of the article and provided financial support. All authors agree on the journal to which they are submitting their manuscript.

## FUNDING INFORMATION

This study was supported by the National Natural Science Foundation of China (no.: 30971306) and the Novel Clinical Diagnosis and Treatment Technology Research Project of Nantong City (MS 12018036).

## CONFLICT OF INTEREST STATEMENT

The authors declare no conflicts of interest.

## Supporting information


**Table S1.** Normality test for continuous variables.

## Data Availability

These data are available to researchers.
