# Supplementary material for: Impact of sarcopenia on the prognosis of patients with advanced non‐small cell lung cancer treated with antiangiogenic therapy: A propensity score matching analysis
Source: Thorac Cancer. 2024 Sep 22;15(31):2248–59. doi: 10.1111/1759-7714.15443 (PMC11543275; doi:10.1111/1759-7714.15443)
Supplement: Supplementary file 1 — Table S1. Normality test for continuous variables. [file TCA-15-2248-s001.pdf]

TableS1.Normality test for continuous variables.

Characteristics	Test-value	P-value
Age (years)[a]	63.71 ± 9.50	0.107
ALT (IU/L)[b]	20(14, 29)	<0.001*
AST (IU/L)[b]	24(20, 32)	<0.001*
ALP (IU/L)[b]	84(67, 111)	<0.001*
LDH (IU/L)[b]	236(190, 310)	<0.001*
Total bilirubin (μmol/L)[b]	9.4(7.25, 12.6)	<0.001*
Direct bilirubin (μmol/L)[b]	2.3(1.5, 3.6)	<0.001*
D-dimer (mg/L)[b]	0.75(0.445, 1.61)	<0.001*
PT (sec)[b]	11.4(10.9, 12.1)	<0.001*
APTT (sec)[b]	28.1(26.75, 29.45)	<0.001*
BUN (mmol/L)[b]	5.7(4.75, 7.05)	<0.001*
Cr (μmol/L)[b]	68(57, 77.5)	<0.001*
WBC (10^9/L)[b]	5.8(4.75, 7.5)	<0.001*
Hemoglobin (g/L)[a]	119.94 ± 18.99	0.618
PLT (10^9/L)[b]	202(157.5, 258.5)	<0.001*
Neutrophils (10^9/L)[b]	3.69(2.77, 5.13)	<0.001*
Lymphocyte (10^9/L)[b]	1.29(0.95, 1.56)	<0.001*
Total cholesterol (mmol/L)[b]	4.3(3.7, 4.8)	<0.001*
NLR[b]	2.97(2.06, 4.55)	<0.001*
PLR[b]	163.33(116.2, 224.33)	<0.001*
Albumin (g/L)[a]	39.02 ± 5.19	0.16
Globulin (g/L)[b]	29.3(26.8, 32.9)	<0.001*
A/G[b]	1.33(1.13, 1.48)	<0.001*
Serum CEA(ng/mL)[b]	8.7(3.5, 39.95)	<0.001*
Triglycerides (mmol/L)[b]	1.33(0.96, 1.71)	<0.001*
HDL (mmol/L)[b]	1.06(0.89, 1.26)	<0.001*
LDL (mmol/L)[a]	2.75 ± 0.74	0.296

Continuous variables[a] with normal distribution are presented as mean value ±SD while others[b] are presented as median (IQR). Categorical variables are presented as frequency(percentage) unless otherwise stated.
